# Lipid-regulated degradation of HMG-CoA reductase and Insig-1 through distinct mechanisms in insect cells

**DOI:** 10.1194/jlr.M033639

**Published:** 2013-04

**Authors:** Rebecca A. Faulkner, Andrew D. Nguyen, Youngah Jo, Russell A. DeBose-Boyd

**Affiliations:** Howard Hughes Medical Institute, Department of Molecular Genetics, University of Texas Southwestern Medical Center, Dallas, TX 75390-9046

**Keywords:** endoplasmic reticulum-associated degradation, lipid metabolism, ubiquitin ligase, cytosolic dislocation, 3-hydroxy-3-methylglutaryl-CoA

## Abstract

In mammalian cells, levels of the integral membrane proteins 3-hydroxy-3-methylglutaryl-CoA reductase and Insig-1 are controlled by lipid-regulated endoplasmic reticulum-associated degradation (ERAD). The ERAD of reductase slows a rate-limiting step in cholesterol synthesis and results from sterol-induced binding of its membrane domain to Insig-1 and the highly related Insig-2 protein. Insig binding bridges reductase to ubiquitin ligases that facilitate its ubiquitination, thereby marking the protein for cytosolic dislocation and proteasomal degradation. In contrast to reductase, Insig-1 is subjected to ERAD in lipid-deprived cells. Sterols block this ERAD by inhibiting Insig-1 ubiquitination, whereas unsaturated fatty acids block the reaction by preventing the protein's cytosolic dislocation. In previous studies, we found that the membrane domain of mammalian reductase was subjected to ERAD in *Drosophila* S2 cells. This ERAD was appropriately accelerated by sterols and required the action of Insigs, which bridged reductase to a *Drosophila* ubiquitin ligase. We now report reconstitution of mammalian Insig-1 ERAD in S2 cells. The ERAD of Insig-1 in S2 cells mimics the reaction that occurs in mammalian cells with regard to its inhibition by either sterols or unsaturated fatty acids. Genetic and pharmacologic manipulations coupled with subcellular fractionation indicate that Insig-1 and reductase are degraded through distinct mechanisms that are mediated by different ubiquitin ligase complexes. Together, these results establish *Drosophila* S2 cells as a model system to elucidate mechanisms through which lipid constituents of cell membranes (i.e., sterols and fatty acids) modulate the ERAD of Insig-1 and reductase.

In mammalian cells, the endoplasmic reticulum-associated degradation (ERAD) pathway controls levels of two integral membrane proteins that play important roles in the maintenance of lipid homeostasis, 3-hydroxy-3-methylglutaryl-CoA (HMG-CoA) reductase and Insig-1. HMG-CoA reductase catalyzes the reduction of HMG-CoA to mevalonate, a rate-limiting reaction in the synthesis of cholesterol and essential nonsterol isoprenoids (1). Sterol accumulation triggers binding of reductase to either Insig-1 or its highly related isoform Insig-2 in endoplamic reticulum (ER) membranes (2–5). Insig binding is mediated entirely by the membrane domain of reductase, which contains eight membrane-spanning helices and precedes a large C-terminal cytosolic domain containing all of the enzyme's catalytic activity (6, 7). Insigs associate with two membrane-bound ubiquitin ligases called gp78 and Trc8 that initiate ubiquitination of reductase (8). This ubiquitination marks reductase for membrane extraction and dislocation into the cytosol for proteasomal degradation through a reaction mediated by the AAA-ATPase VCP/p97 (9).

Insigs also mediate the sterol regulation of Scap (10), an ER membrane protein that like reductase contains an N-terminal membrane domain with eight membrane-spanning helices, followed by a cytosolic C-terminal domain. The C-terminal domain of Scap mediates an association with membrane-bound transcription factors called sterol regulatory element-binding proteins (SREBPs) (11). In sterol-deprived cells, Scap mediates translocation of SREBPs from the ER to the Golgi where transcriptionally active fragments of SREBPs become proteolytically released from Golgi membranes (12–14). These fragments then migrate into the nucleus and activate transcription of target genes, which include reductase and other enzymes required for synthesis of cholesterol and fatty acids (15). Sterol-induced binding of Insigs to the membrane domain of Scap traps the protein along with its associated SREBP in the ER (16). Without transport to the Golgi, SREBPs do not become proteolytically activated and expression of SREBP target genes declines.

Topology studies indicate that the mammalian Insig proteins consist of six transmembrane helices separated by short hydrophilic loops (10). Although the membranous regions of Insig-1 and Insig-2 exhibit 85% amino acid identity and both proteins bind to Scap and reductase in a sterol-regulated manner, only Insig-1 is subjected to ERAD (17). In contrast to that of reductase, the ERAD of Insig-1 is inhibited by sterols and the reaction is also blocked by unsaturated fatty acids (17). When cells are deprived of lipids (i.e., cholesterol and fatty acids), Insig-1 binds to gp78 rather than to reductase or Scap and thus becomes ubiquitinated and degraded. Sterol-induced binding of Insig-1 to Scap displaces gp78 and thereby prevents Insig-1 ubiquitination and degradation. Unsaturated fatty acids do not block Insig-1 ubiquitination, but they rather prevent the protein's ERAD by inhibiting its association with the ubiquitin regulatory X (Ubx) domain containing protein-8 (Ubxd8), which mediates recruitment of VCP/p97 to membranes (18, 19). Thus, unsaturated fatty acids inhibit the ERAD of Insig-1 by blocking its membrane extraction into the cytosol.

Although membrane extraction and cytosolic dislocation are well-established events in the ERAD of integral membrane proteins such as Insig-1 and reductase (20), underlying mechanisms for these reactions are not fully understood. To accelerate discovery of additional factors that mediate ERAD of integral membrane proteins, we previously examined sterol-accelerated ERAD of mammalian reductase in *Drosophila* S2 cells (21). We chose to study reductase ERAD in S2 cells because they lack a recognizable Insig gene and cannot synthesize sterols de novo (22, 23). In addition, general ERAD components are highly conserved from yeast to humans (see **Table 1**) (24). Thus, the potential role of these components in reductase ERAD can be readily determined in RNA interference (RNAi) experiments, which can be effectively executed in S2 cells (25). Our initial studies revealed that in S2 cells ERAD of the membrane domain of mammalian reductase, the minimal requirement for sterol-accelerated ERAD (2, 7), precisely mirrored the reaction that occurs in mammalian cells with regard to: *i*) dependence on the action of mammalian Insig-1 or Insig-2; *ii*) maximal stimulation by sterols plus nonsterol isoprenoids; and *iii*) inhibition by the proteasome inhibitor MG-132 (21). The *Drosophila* homolog of the yeast ubiquitin ligase Hrd1 (designated dHrd1), which exhibits significant sequence homology with gp78, was found to be required for sterol-accelerated reductase ERAD in S2 cells. These findings suggest that mechanisms for Insig-dependent ERAD of reductase and factors that mediate these reactions are highly conserved in *Drosophila* S2 cells.

**TABLE 1.** Components of the ER-associated degradation pathway

*S. cerevisiae*	Mammalian	*Drosophila*
Hrd1	Hrd1, gp78	dHrd1*^a^*
Doa10	Teb4	dTeb4
	Trc8	dTrc8
Ubc6	Ubc6	dUbc6
Ubc7	Ube2g2 (Ubc7)	dUbc7*^a^* (courtless)
Hrd3	Sel1	dSel1*^a^* (dHrd3)
Yos9	Os9, XTP3-B	dOs-9*^a^*
Kar2	Bip	dHsc70*^a^*
Usa1	Herp	dHerp*^a^*
Der1	Derlin-1, -2, -3	dDerlin-1, -2/3*^a^*
Ubx2	Ubxd2, Ubxd8	dUbxd2*^a^*, dUbxd8*^a^*
cdc48	VCP/p97	dTer94*^a^*
Npl4	Npl4	dNpl4*^a^*
Ufd1	Ufd1	dUfd1*^a^*
Dsk2	Ubiquilin-1, -2, -3, -4	dUbiquilin*^a^*
Rad23	Rad23	DHR23*^a^*
Ube4a	Ube4a	dUbe4a

a Identified in dHrd1-TAP experiments.

Considering that specificity of substrate ubiquitination is primarily determined by ubiquitin ligases that exist in large multiprotein complexes (24, 26, 27), we initiated the current studies by characterizing the dHrd1 ubiquitin ligase complex in S2 cells. Tandem affinity purification of dHrd1 coupled with mass spectrometry led to the identification of *Drosophila* homologs of several proteins known to associate with Hrd1 in yeast. RNAi together with degradation and cytosolic dislocation assays were subsequently employed to determine a role for these newly identified components of the *Drosophila* ERAD pathway in mammalian reductase degradation. We also reconstituted the ERAD of mammalian Insig-1 in S2 cells and found that the reaction was regulated by both sterols and unsaturated fatty acids through similar mechanisms that occur in mammalian cells. Further investigation revealed that while reductase ERAD was mediated by dHrd1 in S2 cells, the ERAD of Insig-1 required another *Drosophila* ubiquitin ligase called dTeb4. The membrane-bound dTeb4 is a close homolog of mammalian Teb4 and yeast Doa10 (28). Remarkably, dHrd1 and dTeb4 degraded reductase and Insig-1 through completely distinct mechanisms. The reductase appeared to become ubiquitinated on ER membranes prior to its dislocation into the cytosol and proteasomal degradation. In contrast, Insig-1 became dislocated into the cytosol prior to its ubiquitination in a manner similar to that proposed for soluble ERAD substrates (29). Considered together, these results not only establish *Drosophila* S2 cells as a viable model system to elucidate general mechanisms for lipid-mediated ERAD of reductase and Insig-1, but they also reveal that ubiquitin ligases can dictate the ERAD pathway through which integral membrane substrates become degraded.

## MATERIALS AND METHODS

### Materials

We obtained cycloheximide, oleate, and 25-hydroxycholesterol from Sigma; fatty acid-free BSA from Roche Molecular Biochemicals; blasticidin from Invitrogen; MG-132 from Peptide Institute, Inc. (Osaka, Japan); digitonin from Calbiochem; Fos-choline-13 from Anatrace; anti-Myc-coupled agarose beads from Sigma; and PYR-41 from Boston Biochem. Stock solutions of oleate were prepared in 0.15 M NaCl and 10% (w/v) fatty acid-free BSA as previously described (30). Other reagents, including sodium mevalonate, lipoprotein-deficient serum (LPDS), and delipidated fetal calf serum were prepared as previously described (30, 31).

### Expression plasmids

The following expression plasmids have been previously described in the indicated reference: pAc-HMG-Red-T7 (TM1-8), which encodes the membrane domain (amino acids 1–346) of hamster reductase fused to three copies of the T7 epitope under transcriptional control of the *Drosophila* actin 5c promoter (pAc) (21); pAc-Insig-1-Myc and pAc-Insig-2-Myc encoding amino acids 1–277 and 1–225 of human Insig-1 and -2, respectively, followed by six copies of the c-Myc epitope (23); pAc-Scap encoding amino acids 1–1,276 of hamster Scap (23); and pAc-dHrd1-T7 encoding amino acids 1–626 of *Drosophila* Hrd1 (21). The pAc-dHrd1-tandom affinity purification (TAP) expression plasmid was generated by replacing the T7 epitope in pAc-dHrd1-T7 with three copies of the FLAG epitope followed by a cleavage site for the tobacco etch virus (TEV) protease and Protein A. The open reading frame for the *Drosophila* homolog of Teb4 (designated dTeb4, CG1317) was amplified by PCR with the Phusion DNA Polymerase Kit (New England Biolabs) using first strand cDNA obtained by reverse transcription of total RNA isolated from S2 cells. Primers used in this amplification contained sequences that encode for a single epitope derived from human influenza hemagglutinin (HA). The PCR products were gel purified, subjected to restriction enzyme digest, and subcloned into the pAc5.1/V5-HisB expression vector. The QuikChange™ Site-Directed Mutagenesis Kit (Stratagene) was used to mutate cysteine-10 in pAc-dTeb4-HA to serine, creating a catalytically inactive RING finger mutant of the enzyme. The pAc-HA-ubiquitin expression plasmid was obtained by cloning the cDNA for human ubiquitin containing a single N-terminal HA epitope into the pAc5.1/V5-HisB expression vector. The integrity of all plasmids was confirmed by DNA sequencing.

### Culture and transfection of *Drosophila* S2 cells

Stock cultures of *Drosophila* S2 cells were maintained in a monolayer in medium A (Schneider's *Drosophila* medium) supplemented with 10% (v/v) heat-inactivated fetal calf serum (HI-FCS) at 23°C. The cells were set up for experiments in 6-well plates on day 0 at a density of 1 × 10^6^ cells per well in medium A supplemented with 10% HI-FCS. On day 1 the cells were washed with medium B (Express Five Serum Free Medium) and transfected with 0.03–3 μg of DNA/well using Maxfect™ Transfection Reagent (KD Medical) at a ratio of 1 μg DNA to 5 μl Maxfect™ in 1 ml of medium B. The total amount of DNA transfected per well was kept constant in each experiment by the addition of empty pAc5.1 vector. On day 2, each well received 1 ml of medium C (Schneider's *Drosophila* medium containing 100 units/ml penicillin and 100 μg/ml streptomycin sulfate) supplemented with 20% HI-FCS, heat-inactivated lipoprotein-deficient serum (HI-LPDS), or heat-inactivated delipidated fetal calf serum (HI-DFCS) (10% final concentration). Following incubation for 24 h at 23°C, cells were subjected to treatments described in figure legends and harvested for analysis as described below.

### Stable transfection of *Drosophila* S2 cells

*Drosophila* S2 cells were set up in 6-well plates on day 0 at a density of 1 × 10^6^ cells per well in medium A supplemented with 10% HI-FCS. On day 1, cells were washed with medium B and transfected with 1 μg of pAc-dHrd1-TAP together with 50 ng of pCoBlast selection vector in medium B using Maxfect™. On day 2, each well received 1 ml of medium C supplemented with 20% HI-FCS (final concentration 10%). Selection began on day 3 by refeeding cells with medium C containing 10% HI-FCS and 5 μg/ml blasticidin. Medium was changed as needed until colonies formed. Single cell colonies were isolated and screened for expression of dHrd1-TAP by immunoblotting detergent lysates with anti-FLAG IgG. A single colony of cells (designated S2/dHrd1-TAP) was selected and maintained in medium A supplemented with 10% (v/v) HI-FCS and 5 μg/ml blasticidin.

### Tandem affinity purification

S2/dHrd1-TAP cells grown in suspension flasks were collected by centrifugation at 1,500 *g* for 5 min at 4°C. Cell pellets were washed with PBS and lysed in buffer containing 10 mM HEPES-KOH pH 7.4, 10 mM KCl, 1.5 mM MgCl_2_, 5 mM EDTA, 5 mM EGTA, 5 mM dithiothreitol, and 0.1 mM leupeptin supplemented with 1% digitonin and a protease inhibitor cocktail (25 μg/ml *N*-acetyl-leucinal-leucinal-norleucinal, 2 μg/ml aprotinin, 0.5 mM Pefabloc, 5 μg/ml pepstatin A, 0.5 mM phenylmethylsulfonyl fluoride). Clarified lysates were subjected to immunoprecipitation with human IgG-conjugated Sepharose beads (GE Healthcare) for 16 h at 4°C. After washing the immunoprecipitates 5 times (15 min each) in lysis buffer containing 0.1% digitonin, precipitated proteins were eluted from the beads by treatment with AcTEV protease (Invitrogen) for 16 h at 4°C. The released proteins were subjected to a second round of immunoprecipitation with anti-FLAG-coupled agarose beads (Sigma) for 5 h at 4°C. Following extensive washes in lysis buffer containing 0.1% digitonin, bound proteins were eluted by rotating the beads with a peptide containing 5 copies of the FLAG epitope (custom synthesized by Genemed Synthesis). The eluted material was subsequently fractionated by SDS-PAGE and the proteins were visualized by Colloidal Blue (Invitrogen) staining. Segments of the gel that contained visible bands were excised and proteins were identified by tandem mass spectroscopy in the Protein Chemistry Core Facility at the University of Texas Southwestern Medical Center.

### Preparation of whole cell lysates

Treatment conditions prior to harvest are described in the figure legends. Following treatments, cells from triplicate wells were combined and collected by centrifugation at 1,500 *g* for 5 min at 4°C. Cell pellets were washed with PBS and resuspended in buffer containing 50 mM Tris-HCl pH 8.0, 150 mM NaCl, 0.1% (w/v) SDS, 1.5% (w/v) Nonidet P-40, 0.5% (w/v) sodium deoxycholate, and 2 mM MgCl_2_ supplemented with the protease inhibitor cocktail. The cell suspension was then lysed by passage through a 22-gauge needle and subsequently rotated for 30 min at 4°C. Insoluble material was removed by centrifugation at 17,000 *g* for 15 min at 4°C and clarified lysates were mixed with SDS-PAGE loading buffer.

### Subcellular fractionation

Following treatments described in the figure legends, cells from triplicate wells were scraped, washed in PBS, and the cell pellet resuspended in buffer containing 10 mM HEPES-KOH pH 7.4, 10 mM KCl, 1.5 mM MgCl_2_, 5 mM EDTA, 5 mM EGTA, 5 mM dithiothreitol, 0.1 mM leupeptin, and 250 mM sucrose supplemented with the protease inhibitor cocktail. The cell suspension was passed through a 22-gauge needle and centrifuged at 1,000 *g* for 7 min at 4°C. The resulting postnuclear supernatants were further subjected to centrifugation at 100,000 *g* for 1 h at 4°C. The pellet fraction obtained from this spin (designated membranes) was resuspended in buffer containing 10 mM Tris-HCl pH 6.8, 100 mM NaCl, 1% (w/v) SDS, 1 mM EDTA, and 1 mM EGTA, and mixed with SDS-PAGE loading buffer. The supernatant fraction obtained from the 100,000 *g* spin (designated cytosol) was precipitated overnight with 5× volume of acetone at −20°C; precipitated material was pelleted by centrifugation at 17,000 *g* for 10 min at 4°C and resuspended in buffer containing 10 mM Tris-HCl pH 6.8, 100 mM NaCl, 1% (w/v) SDS, 1 mM EDTA, and 1 mM EGTA, and subsequently mixed with SDS-PAGE loading buffer.

### Immunoblot analysis and immunoprecipitation of Insig-1

Aliquots of whole cell lysates, membrane, or cytosol fractions were subjected to 10% SDS-PAGE after which the proteins were transferred to nitrocellulose membranes (GE Healthcare). Immunoblot analysis was carried out with the following primary antibodies: monoclonal anti-T7 Tag IgG (Novagen), IgG-9E10, a mouse monoclonal antibody against the c-Myc epitope purified from culture medium of hybridoma clone 9E10 (American Type Culture Collection), IgG-3B2, a mouse monoclonal antibody against *Drosophila* SREBP (32), IgG-9D5, a mouse monoclonal antibody against hamster Scap (33), monoclonal anti-HA IgG (Sigma), polyclonal anti-actin IgG (Sigma), and anti-E1 IgG (Calbiochem). Primary antibodies were detected with horseradish peroxidase-conjugated donkey anti-mouse, anti-rabbit, or anti-biotin IgG ( Jackson ImmunoResearch Laboratories) using SuperSignal West Pico Chemiluminescent Substrate (Thermo Scientific).

Immunoprecipitation of transfected Insig-1-Myc from detergent lysates of S2 cells was carried out as previously described (17). Briefly, cells were harvested, lysed in PBS containing 1% Fos-choline-13 and subjected to centrifugation at 17,000 *g* for 15 min at 4°C. The clarified lysates were adjusted to 2 M urea and immunoprecipitated with 100 µl anti-Myc-coupled agarose beads. Aliquots of the immunoprecipitates were then subjected to SDS-PAGE followed by immunoblot analysis with monoclonal anti-HA IgG (against ubiquitin) and IgG-9E10 (against Insig-1).

### Production of double-stranded RNA

Total RNA isolated from *Drosophila* S2 cells using RNA STAT 60 (Tel-Test, Inc.) was subjected to reverse transcription PCR using the TaqMan® reagents (Applied Biosystems). DNA templates for double-stranded RNA (dsRNA) synthesis were amplified from first strand cDNA using the Phusion DNA polymerase (New England Biolabs) and previously described primers (21). The resulting PCR products were purified using the QIAquick PCR Purification Kit (Qiagen) and used as templates to generate dsRNAs using the MEGAscript® T7 Kit (Ambion). Resulting dsRNAs were purified from the reaction using the RNeasy Mini Kit (Qiagen).

### RNA interference-mediated knockdown in *Drosophila* S2 cells

S2 cells were plated on day 0 in 6-well plates at a density of 1 × 10^6^ cells/well in 1 ml of medium B. Immediately after plating, 15 μg of dsRNA was added to each well and incubated for 1 h. Each well subsequently received 2 ml of medium C supplemented with either 10% HI-FCS, HI-LPDS, or HI-DFCS.

### Isolation of total RNA and quantitative real-time PCR analysis

The total RNA isolated from S2 cells using STAT 60 was subjected to reverse transcription PCR as described above. Quantitative real-time PCR was performed as previously described (21, 34). The comparative C_t_ method was used to calculate the relative expression and the *Drosophila* ribosomal protein 49 was used as an internal control to account for variations in mRNA levels.

## RESULTS

In previous studies, we found that the *Drosophila* homolog of the yeast *Saccharomyces cerevisiae* ubiquitin ligase Hrd1 plays a major role in sterol-accelerated ERAD of mammalian reductase in S2 cells (21). In yeast, Hrd1 exists in a large multiprotein complex that includes its cofactor Hrd3, the cytosolic ubiquitin-conjugating enzyme Ubc7 and its membrane receptor Cue1p, polytopic Derlin-1 and its recruitment factor Usa1, the AAA-ATPase cdc48 and its membrane anchor ubiquitin regulatory-X (ubx) domain-containing protein Ubx2, and the Hsp70 chaperone Kar2 bound to the lectin Yos9 (24). Importantly, all of these factors, except for Cue1p, are highly conserved in mammals (24) (Table 1). It is important to note that Hrd1 mediates regulated ERAD of the reductase isoform Hmg2p in yeast (35). However, sterols do not appear to be the major signal for Hrd1-mediated degradation of Hmg2p, and the reaction is inhibited by the yeast Insig protein (36).To identify proteins that associate with *Drosophila* Hrd1 (dHrd1), we utilized a line of S2 cells that stably overexpress the enzyme fused to a C-terminal TAP tag. The TAP tag is composed of three copies of the FLAG epitope and Protein A separated by a cleavage site for the TEV protease. Detergent lysates of S2 cells overexpressing dHrd1-TAP were subjected to affinity chromatography using IgG- and anti-FLAG-coupled agarose beads. Eluted proteins were fractionated by SDS-PAGE, visualized by Colloidal Blue staining, and identified by mass spectrometry. Precipitation of dHrd1-TAP led to the recovery of *Drosophila* homologs of known components of the yeast Hrd1 complex including Hrd3 (dSel1), Yos9 (dOs9), Kar2 (dHsc70), Usa1 (dHerp), VCP/p97 (Ter94), Ubx2 (dUbxd8 and dUbxd2), Npl4 (dNpl4), Ufd1 (dUfd1), and Der1 (dDerlin-2/3) (Table 1). In addition, several chaperones, lectins, subunits of the proteasome and associated proteins, and other components of the ubiquitin/proteasome pathway were identified in the dHrd1-TAP immunoprecipitation.

**Fig. 1.**
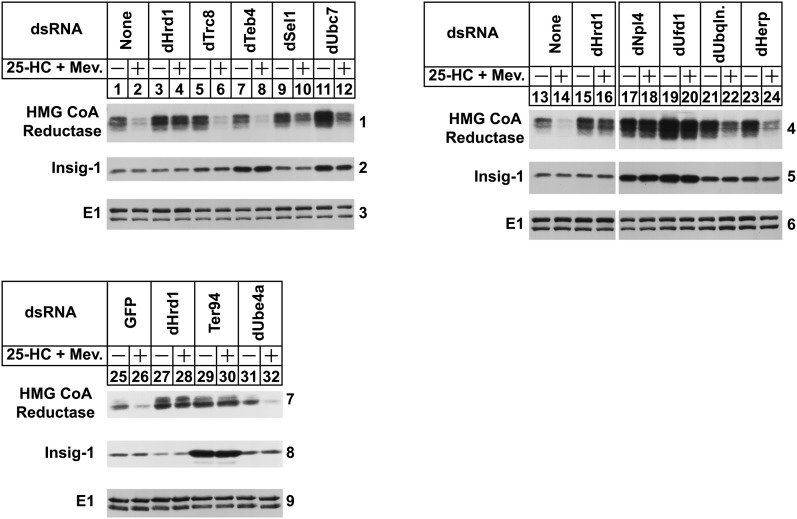
Components of the ERAD pathway required for proteasomal degradation of hamster HMG-CoA reductase in *Drosophila* S2 cells. S2 cells were set up on day 0 in 6-well plates at a density of 1 × 10^6^ cells per well in medium B. Immediately after plating, cells were incubated with 15 μg of dsRNA targeted against the indicated endogenous mRNAs. Following incubation for 1 h, the cells received 2 ml of medium C supplemented with 10% HI-LPDS. On day 1, cells were washed and transfected in medium B with 0.5 μg of pAc-HMG-Red-T7 (TM1-8) and 0.1 μg pAc-Insig-1-Myc in medium B using Maxfect™ Transfection Reagent as described in Materials and Methods. On day 2, each well received 1 ml of medium B supplemented with 20% HI-LPDS. Cells were treated on day 3 with medium C supplemented with 10% HI-LPDS in the absence or presence of 2.5 μM 25-HC plus 10 mM mevalonate (Mev.). Following incubation for 4 h, cells were harvested and lysed in detergent-containing buffer; aliquots of the resulting lysates (50 μg of protein/lane) were separated by 10% SDS-PAGE, the proteins were transferred to nitrocellulose membranes, followed by immunoblot analysis with anti-T7 IgG (against reductase), IgG-9E10 (against Insig-1), and anti-E1 IgG. The numbers to the side of immunoblots are referred to as “panels” in the text. Immunoblots shown in panels 4–6 were cropped from the same gel exposed to film for an identical period of time.

In the experiment of **Fig. 1**, S2 cells were subjected to RNAi-mediated knockdown through incubation with dsRNAs targeting the genes encoding dHrd1, *Drosophila* homologs of two unrelated membrane-bound ubiquitin ligases (dTeb4 and dTrc8), or various proteins identified in the dHrd1 TAP experiment (see Table 1). The cells were then transfected with an expression plasmid encoding the entire membrane domain of hamster reductase tagged with three tandem copies of the T7 epitope together with a plasmid encoding Myc-tagged human Insig-1. Previously, we found that the membrane domain of mammalian reductase is both necessary and sufficient for Insig-mediated sterol-accelerated ERAD in S2 as well as mammalian cells (2, 21). Following transfection, cells were treated in the absence or presence of the oxysterol 25-hydroxycholesterol (25-HC) plus mevalonate to maximally stimulate reductase ERAD (3, 37–39). The cells were subsequently harvested and lysed in detergent-containing buffer. Immunoblot analysis of the resulting lysates with anti-T7 antibody revealed that levels of the membrane domain of reductase were reduced upon treatment of control-treated cells with 25-HC plus mevalonate (Fig. 1, panels 1, 4, and 7, lanes 2, 14, and 26), indicating accelerated ERAD. Consistent with our previous results (21), RNAi-mediated knockdown of dHrd1 (panels 1, 4, and 7, lanes 4, 16, and 28) or its associated proteins dSel1, dUbc7, dNpl4, dUfd1, and dHerp (lanes 10, 12, 18, 20, and 24) slowed reductase ERAD. Knockdown of dUbiquilin, the *Drosophila* homolog of the yeast protein Dsk2p, or Ter94, the VCP/p97 homolog, also inhibited the reaction (panels 4 and 7, lanes 22 and 30). In contrast, sterol-accelerated ERAD of reductase continued in dTeb4 knockdown cells (panel 1, lane 8). Similarly, knockdown of the ubiquitination factor dUbe4a (panel 7, lane 32), DHR23 (yeast Rad23 homolog), or the chaperone dHsc70, failed to block reductase ERAD in S2 cells (data not shown). Insig-1 was stabilized by certain dsRNA treatments (panels 2, 5, and 8); the significance of these effects will be addressed below. Levels of the loading control, E1, remained constant throughout the RNAi experiments (panels 3, 6, and 9). Quantitative real-time PCR revealed that target gene expression was reduced 70–95% by RNAi. Unfortunately, we could not determine protein levels in these experiments owing to the lack of antibodies capable of recognizing the various *Drosophila* proteins.

**Fig. 2.**
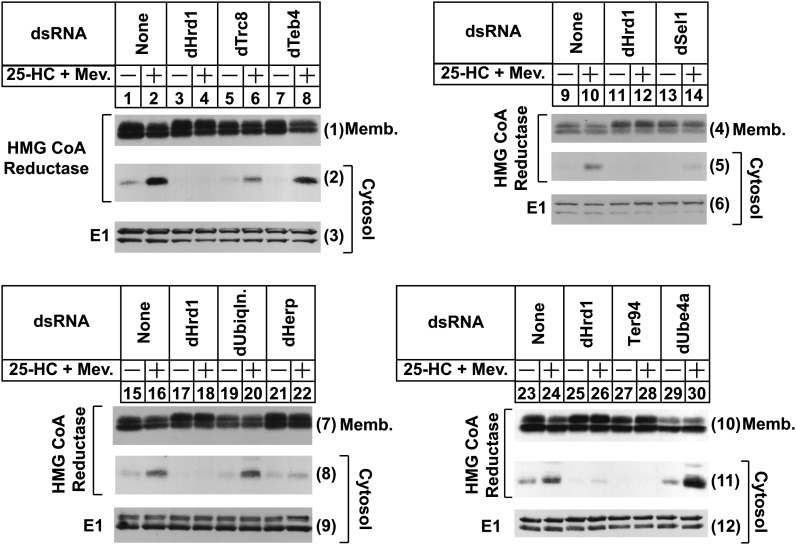
ERAD components required for sterol-induced cytosolic dislocation of hamster HMG-CoA reductase in *Drosophila* S2 cells. S2 cells were set up for experiments and treated with dsRNA on day 0, transfected with pAc-HMG-Red-T7 (TM1-8) and pAc-Insig-1-Myc on day 1, incubated with medium B containing 10% LPDS on day 2, and subjected to treatment with 25-HC plus mevalonate (Mev.) on day 3 as described in the legend to Fig. 1. The cells also received 10 µM MG-132 on day 3. Following incubation for 4 h, the cells were harvested and subjected to subcellular fractionation as described in Materials and Methods. The resulting membrane (Memb.) (10 μg protein/lane) and cytosol (40 μg protein/lane) fractions were subjected to 10% SDS-PAGE followed by immunoblot analysis as described in the legend to Fig. 1. The numbers to the side of immunoblots are referred to as panels in the text.

Our previous studies in mammalian cells indicated that following ubiquitination, reductase becomes dislocated from ER membranes into the cytosol prior to proteasome-mediated degradation (9). Considering this, we next designed an experiment to determine the genetic requirements for cytosolic dislocation of the membrane domain of reductase in S2 cells. In **Fig. 2**, S2 cells subjected to RNAi and transfected with the membrane domain of reductase and Insig-1 were treated with the proteasome inhibitor MG-132 (to block degradation of any dislocated reductase) in the absence or presence of 25-HC plus mevalonate. Following treatments, the cells were harvested and lysed in the absence of detergents for the preparation of postnuclear supernatants that were subjected to centrifugation at 100,000 *g*. The resulting pellet and supernatant fractions (designated membranes and cytosol, respectively) were then analyzed by immunoblot. The results show that 25-HC plus mevalonate enhanced the appearance of the membrane domain of reductase in the cytosol fraction of transfected S2 cells (Fig. 2, panels 2, 5, 8, and 11, lanes 2, 10, 16, and 24). Knockdown of dHrd1 led to the reduced appearance of the protein in the cytosol of 25-HC plus mevalonate-treated cells (panels 2, 5, 8, and 11, lanes 4, 12, 18, and 26). However, reductase continued to become dislocated into the cytosol of dTrc8 (panel 2, lane 6) and dTeb4 (panel 2, lane 8) knockdown cells. Knockdown of dSel1 (panel 5, lane 14), dHerp (panel 8, lane 22), and Ter94 (panel 11, lane 28) blunted sterol-induced cytosolic dislocation of reductase. Knockdown of dTrc8 slightly blocked reductase dislocation (panel 1, lane 6), which may reflect a minor secondary role in the reaction. Cytosolic dislocation of reductase continued in dUbiquilin (panel 8, lane 20) as well as in dUbe4a (panel 11, lane 30) knockdown cells.

**Fig. 3.**
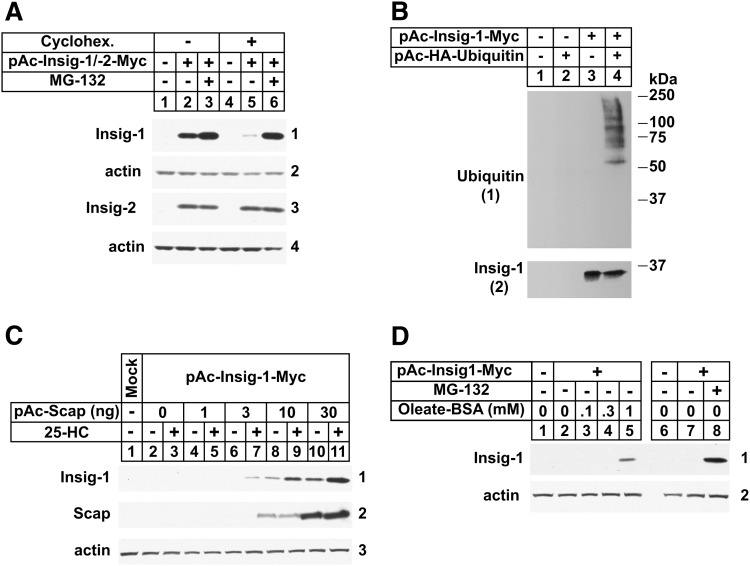
Reconstitution of lipid-regulated ERAD of mammalian Insig-1 in *Drosophila* S2 cells. S2 cells were set up in 6-well plates on day 0 at 1 × 10^6^ cells per well in medium A supplemented with 10% HI-FCS. On day 1, cells were transfected in medium B using Maxfect™ as follows. A: 0.1 µg of pAc-Insig-1-myc or 0.1 µg of pAc-Insig-2-myc. B: 0.2 µg pAc-Insig-1-Myc in the absence or presence of 1.0 µg pAc-HA-ubiquitin. C: 0.1 µg of pAc-Insig-1-myc and 1, 3, 10, or 30 ng of pAc-Scap. D: 0.1 of µg pAc-Insig-1-myc. Total amount of DNA was adjusted to 0.1 μg (A, D), 1.2 μg (B), or 0.13 μg (C) using empty pAc5.1 vector. On day 2, each well received 1 ml of medium B supplemented with 20% HI-LPDS (A–C) or HI-DFCS (D). Cells were treated on day 3 with medium C supplemented with 10% HI-LPDS (A–C) or HI-DFCS (D) under the following conditions. A: In the absence or presence of 10 μM MG-132 (6 h) and 50 μM cycloheximide (2 h). B: In the presence of 10 µM MG-132 (2 h). C: In the absence and presence of 2.5 μM 25-HC and 10 mM mevalonate (4 h) together with 50 μM cycloheximide (2 h). D: In the absence and presence of 10 μM MG-132 (6 h) or 0.1, 0.3, or 1 mM BSA-oleate (4 h) together with 50 μM cycloheximide (2 h). A, C, D: Following incubations, cells were harvested and aliquots of whole cell lysates [30 μg protein/lane (A), 40 μg protein/lane (C), 50 μg protein/lane (D)] were subjected to 10% SDS-PAGE followed by immunoblot analysis with IgG-9E10 (against Insigs), IgG-9D5 (against hamster Scap), and anti-actin IgG. B: Following incubation, the cells were harvested for preparation of detergent lysates that were immunoprecipitated with 60 µl anti-Myc coupled agarose beads. Aliquots of the immunoprecipitates were then subjected to SDS-PAGE followed by immunoblot analysis with anti-HA (against ubiquitin) and IgG-9E10 (against Insig-1). The numbers to the side of immunoblots are referred to as panels in the text.

Despite their high degree of homology, Insig-1, but not Insig-2 is subjected to lipid-regulated ERAD in mammalian cells (17, 19). Considering our successful results with reductase ERAD, we next sought to determine whether physiologically relevant ERAD of mammalian Insig-1 degradation could be reconstituted in S2 cells. **Fig. 3A** compares the expression of Insig-1-Myc and Insig-2-Myc in S2 cells following treatment in the absence or presence of the protein synthesis inhibitor cycloheximide. In the absence of cycloheximide, transfection of S2 cells with the appropriate plasmid led to detectable expression of Myc-tagged Insig-1 and Insig-2 (Fig. 3A, panels 1 and 3, lane 2). MG-132 treatment led to a small but detectable increase in the amount of Insig-1 (panel 1, lane 3), but the amount of Insig-2 remained unchanged (panel 3, lane 3). Expression of Insig-1 was markedly reduced following cycloheximide treatment (Fig. 3A, panel 1, compare lanes 2 and 5). This reduction was completely abolished when the cells were also treated with MG-132 (lane 6), indicating the proteasome-mediated degradation of Insig-1 in the absence of the inhibitor. In contrast, the level of Insig-2 was constant in cycloheximide-treated S2 cells relative to that in untreated cells, regardless of the presence or absence of MG-132 (panel 3, lanes 5 and 6).

We next evaluated the ubiquitination status of Insig-1 in S2 cells. For this purpose, we transfected S2 cells with various combinations of expression plasmids encoding Myc-tagged Insig-1 and HA-tagged ubiquitin, and treated them with MG-132. Following treatments, the cells were harvested; detergent lysates were immunoprecipitated with anti-Myc, followed by immunoblot analysis of precipitated material with anti-HA to visualize ubiquitinated Insig-1. The results show that coexpression of HA-ubiquitin led to the detection of poly-ubiquitinated forms of Insig-1 in the immunoprecipitates (Fig. 3B, panel 1, lane 4).

To determine whether Insig-1 ERAD in S2 cells is subject to lipid-mediated regulation, we began by transfecting cells with Insig-1 and various amounts of hamster Scap and subjected them to treatment with cycloheximide in the absence or presence of 25-HC prior to harvest and lysis. Immunoblot analysis of the lysates revealed that Insig-1 protein was not detectable when expressed alone (Fig. 3C, panel 1, lanes 2 and 3) or together with a low level (1 ng) of plasmid encoding hamster Scap (lanes 4 and 5), regardless of the absence or presence of 25-HC. However, a small amount of Insig-1 was detected upon cotransfection of 3 ng of the Scap-encoding plasmid, but only when the cells were also treated with 25-HC (compare lanes 6 and 7). Cotransfection of higher levels of Scap stabilized Insig-1, even in the absence of 25-HC (panel 1, lanes 8 and 10); this stabilization was further enhanced upon treatment with the sterol (lanes 9 and 11). Results of Fig. 3D show that in the absence of Scap coexpression, Insig-1 ERAD in S2 cells was subjected to regulation by the unsaturated fatty acid oleate. Treatment of cells with oleate stabilized Insig-1 (Fig. 3D, panel 1, lane 5), but to a lesser extent than that observed with MG-132 treatment (lane 8). This may reflect differences in the uptake of the two reagents by S2 cells.

**Fig. 4.**
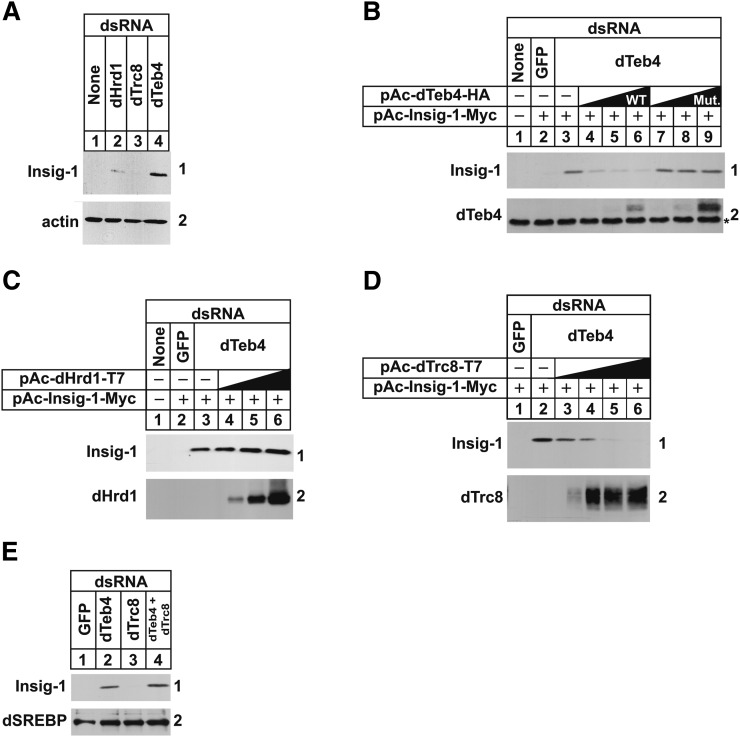
The dTeb4 ubiquitin ligase is required for degradation of mammalian Insig-1 in *Drosophila* S2 cells. S2 cells were set up and subjected to RNAi-mediated knockdown on day 0 and transfected with 0.5 μg of pAc-Insig-1-Myc alone (A, E) or together with 0.1, 0.3, or 1.0 µg of WT or mutant (Mut.) (C10S) pAc-dTeb4-HA (B); 0.1, 0.3, or 1.0 µg of pAc-dHrd1-T7 (C); and 0.1, 0.3, 1.0, or 3.0 μg pAc-dTrc8-T7 (D) on day 1 as described in the legend to Fig. 1. On day 2, each well received 1 ml of medium B supplemented with 20% HI-LPDS. The cells were subsequently switched on day 3 to medium C supplemented with 10% HI-LPDS and 50 μM cycloheximide. Following incubation for 2 h, cells were harvested for preparation of whole cell lysates that were subjected to SDS-PAGE (20 µg protein/lane) and immunoblot analysis with IgG-9E10 (against Insig-1), IgG-3B2 (against dSREBP), anti-HA IgG (against dTeb4), anti-T7 IgG (against dHrd1 and dTrc8), and anti-actin IgG. The asterisk (*) in (B) denotes a nonspecific, cross-reactive band. The numbers to the side of immunoblots are referred to as panels in the text.

To further characterize ERAD of mammalian Insig-1 in S2 cells, we next sought to identify the ubiquitin ligase required for the reaction. S2 cells were subjected first to RNAi-mediated knockdown, after which they were transfected with Insig-1-Myc, treated with cycloheximide, and harvested for preparation of detergent lysates that were analyzed by anti-Myc immunoblot. The results show that Insig-1 continued to become degraded in control cells (**Fig. 4A**, panel 1, lane 1) and in cells treated with dsRNA against mRNAs for dHrd1 and dTrc8 (lanes 2 and 3). However, RNAi-mediated knockdown of the dTeb4 ubiquitin ligase significantly stabilized Insig-1 (lane 4). The specificity of dTeb4 knockdown was evaluated by comparing the ability of wild-type or mutant dTeb4 to restore Insig-1 ERAD in dTeb4 knockdown cells. The mutant form of dTeb4 examined in this experiment harbors a substitution of serine for cysteine-10 in the N-terminal C4HC3 RING domain, which corresponds to cysteine-9 in human Teb4 (28). Mutation of this cysteine residue in human Teb4 abolishes in vitro ubiquitin ligase activity of the enzyme (28). Consistent with results of Fig. 3A, Insig-1 was stabilized in dTeb4 knockdown S2 cells (Fig. 4B, panel 1, compare lanes 2 and 3). Overexpression of wild-type dTeb4 in the knockdown cells restored ERAD of Insig-1 (lanes 4–6), whereas overexpression of the C10S dTeb4 mutant failed to restore the reaction (lanes 7–9). Similarly, overexpression of dHrd1 failed to restore Insig-1 ERAD in dTeb4 knockdown cells (Fig. 4C, panel 1, lanes 4–6), whereas overexpression of dTrc8 unexpectedly restored the reaction (Fig. 4D, panel 1, lanes 3–6). Despite this, endogenous dTrc8 did not appear to contribute to degradation of Insig-1 as indicated by the observation that knockdown of dTrc8 did not appreciably stabilize Insig-1 in dTeb4 knockdown cells (Fig. 4E, panel 1, lanes 2 and 4).

**Fig. 5.**
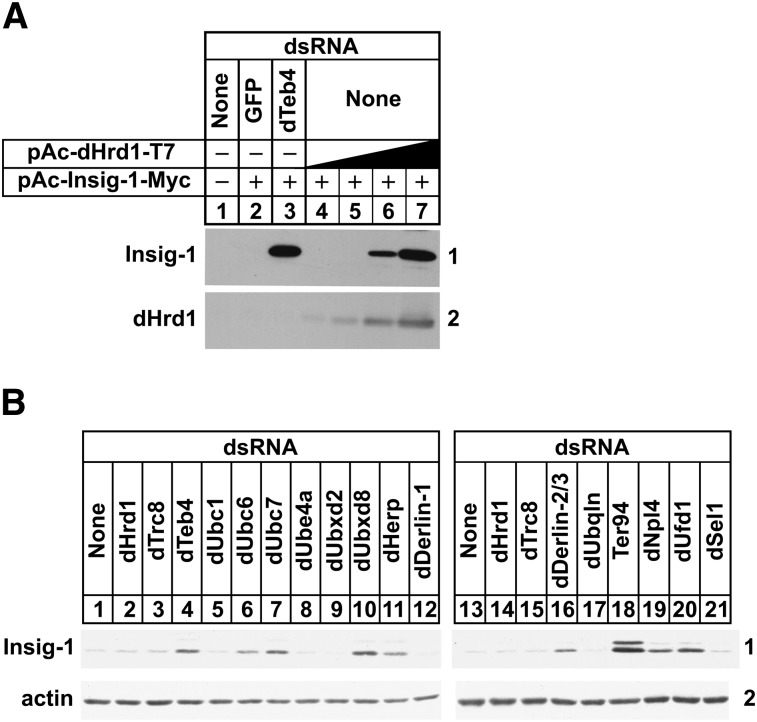
Components of the ERAD pathway required for degradation of mammalian Insig-1 in *Drosophila* S2 cells. S2 cells were set up and subjected to RNAi-mediated knockdown on day 0 and transfected with 0.5 μg of pAc-Insig-1-Myc alone (B) or together with 0.1, 0.3, 1.0, or 3.0 µg of pAc-dHrd1-T7 (A) on day 1 as described in the legend to Fig. 1. On day 2 each well received 1 ml of medium B supplemented with 20% HI-LPDS. On day 3, cells were incubated in medium C supplemented with 10% HI-LPDS and 50 µM cycloheximide. After 2 h, cells were harvested for preparation of detergent lysates that were subjected to SDS-PAGE (20 µg protein/lane) and immunoblot analysis with IgG-9E10 (against Insig-1), anti-T7 IgG (against dHrd1), and anti-actin IgG. The numbers to the side of immunoblots are referred to as panels in the text.

In the absence of RNAi-mediated knockdown, overexpression of dHrd1 inhibited the ERAD of Insig-1 (**Fig. 5A**, panel 1, lanes 4-7). We reasoned that this inhibition resulted from titration of shared ERAD components from the dTeb4 ubiquitin ligase complex. To investigate this notion, we next examined a role for dHrd1 complex components in ERAD of Insig-1 using RNAi. The results of Fig. 5B show that knockdown of dTeb4 as well as dUbc6, dUbc7, dUbxd8, dHerp, dDerlin2/3, Ter94, dNpl4, and dUfd1 significantly blunted Insig-1 ERAD (panel 1, lanes 4, 6, 7, 10, 11, 16, and 18–20).

**Fig. 6.**
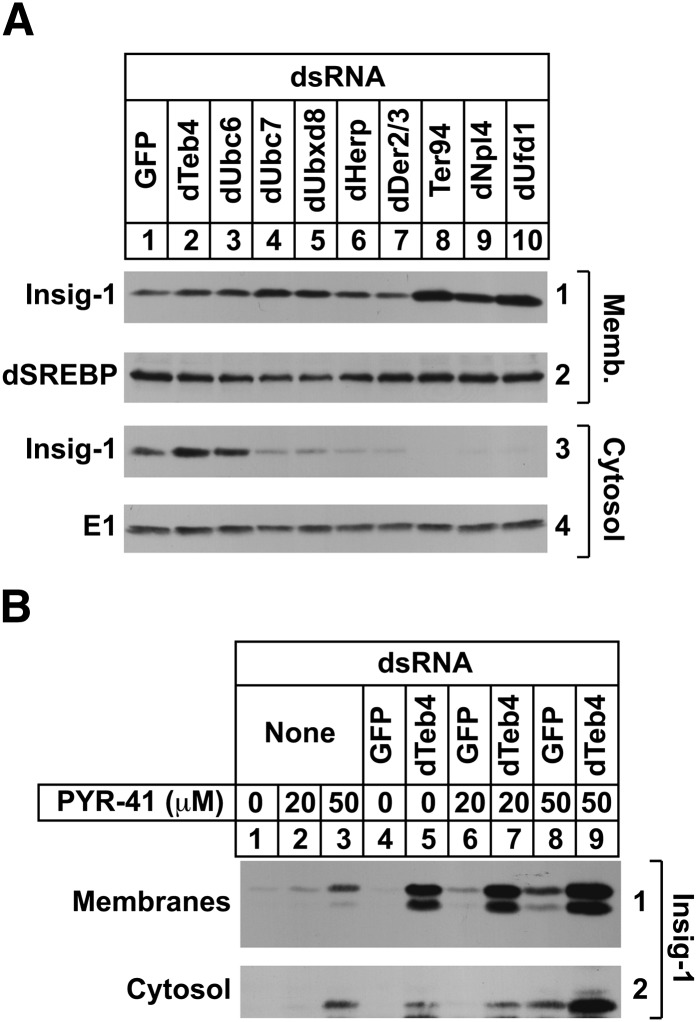
Components of the ERAD pathway required for cytosolic dislocation of mammalian Insig-1 in *Drosophila* S2 cells. S2 cells were set up and subjected to RNAi-mediated knockdown on day 0 and transfected on day 1 with 0.5 µg of pAc-Insig-1-Myc as described in the legend to Fig. 1. On day 2, each well received 1 ml of medium B supplemented with 20% HI-DFCS. On day 3, cells were incubated in medium C containing 10% HI-DFCS, 50 µM cycloheximide, and 10 µM MG-132 (A) or the indicated concentration of PYR-41 (B). Following incubation for 2 h, cells were harvested and subjected to subcellular fractionation. Equal proportions of membrane (Memb.) and cytosol fractions were subjected to SDS-PAGE, followed by immunoblot analysis with IgG-9E10 (against Insig-1), IgG-3B2 (against dSREBP), and anti-actin IgG. The numbers to the side of immunoblots are referred to as panels in the text.

Like reductase, Insig-1 becomes dislocated from ER membranes into the cytosol of mammalian cells for proteasome-mediated ERAD (9). The RNAi experiment of **Fig. 6A** was designed to examine the cytosolic dislocation of mammalian Insig-1 in S2 cells. The results show that a fraction of Insig-1 appeared in the cytosol of MG-132-treated S2 cells that received control GFP dsRNA (Fig. 6A, panel 3, lane 1). This appearance was significantly inhibited by the RNAi-mediated knockdown of dUbc7, dUbxd8, dHerp, dDerlin2/3, Ter94, dNpl4, and dUfd1 (lanes 4–10). Surprisingly, knockdown of dTeb4 did not block the cytosolic dislocation of Insig-1 (lane 2), even though the ubiquitin ligase was found to be required for Insig-1 ERAD (see Fig. 4A). This result suggested that Insig-1 becomes ubiquitinated following its cytosolic dislocation. To further explore this, we next evaluated the effect of the ubiquitin-activating enzyme (E1) inhibitor PYR-41 (40) on Insig-1 dislocation. Treatment with PYR-41 led to an increase in the amount of Insig-1 detected in membranes of transfected S2 cells (Fig. 6B, panel 1, lanes 1–3, 6, and 8), indicating that the inhibitor blocked ERAD of Insig-1. Insig-1 was also stabilized in dTeb4 knockdown cells as expected (lane 5). The combination of dTeb4 knockdown and PYR-41 treatment led to an increased stabilization of Insig-1 in membranes (panel 1, lanes 5–9). Insig-1 also accumulated in the cytosol of cells that were either treated with PYR-41 (Fig. 6B, panel 2, lanes 3 and 8) or subjected to dTeb4 knockdown (lane 5); the combination of dTeb4 knockdown and PYR-41 treatment led to an increased amount of cytosolic Insig-1 in an additive fashion (panel 2, compare lanes 5, 6–9).

## DISCUSSION

The ERAD pathway is an essential, highly conserved process through which misfolded proteins, both soluble within the ER lumen and integral to the ER membrane, are selectively degraded by proteasomes (41). Insights into the underlying mechanisms of ERAD have been traditionally provided through studies of soluble substrates. These studies disclosed that the ERAD pathway can be divided into distinct steps that include substrate recognition by molecular chaperones and ER-resident lectins, retro-translocation across the ER membrane into the cytosol, ubiquitination, and finally, delivery of the ubiquitinated substrate to proteasomes for degradation (29). Although many concepts regarding ERAD of soluble substrates are applicable to integral membrane substrates, a thorough understanding of mechanisms underlying the ERAD of these types of substrates is lacking. In particular, how integral membrane substrates are selected for ERAD and become extracted from membranes during ERAD remains a mystery. Integral membrane ERAD substrates, especially those with multiple membrane-spanning segments, can adopt complex topologies in ER membranes and can potentially present misfolded lesions in the cytosol, the ER lumen, or within the membrane, thereby engaging distinct ERAD pathways (20). Thus, complete elucidation of mechanisms for ERAD of membrane proteins requires rigorous examination of representative model substrates.

HMG-CoA reductase and Insig-1 represent ideal models of integral membrane ERAD substrates. The major virtue of studying reductase and Insig-1 is that their ERAD can be precisely controlled by the addition of sterols and other lipid constituents of cell membranes (i.e., fatty acids and nonsterol isoprenoids). This attribute ensures physiologically relevant ERAD of reductase and Insig-1 when the reactions are reconstituted either in vitro or in model systems. The current study exploits these features by expanding on the previous finding that Insig-mediated sterol-accelerated ERAD of mammalian reductase can be reconstituted in *Drosophila* S2 cells (21). We began by using tandem affinity purification to isolate a multiprotein complex containing the *Drosophila* homolog of the yeast ubiquitin ligase Hrd1 (designated dHrd1), which was previously found to mediate sterol-accelerated reductase ERAD in S2 cells (21). Protein identification by mass spectrometry revealed that the dHrd1 complex contained *Drosophila* homologs of several proteins known to associate with yeast Hrd1 including dSel1, dUbxd8, dUbxd2, Ter94, dNpl4, dUfd1, dUbc7, dDerlin-2/3, dOs9, and dHsc70 (Table 1). In addition, we identified several other components of the ubiquitin/proteasome system including RE16341p (a homolog of mammalian α-mannosidase EDEM3), dUbiquilin, and DHR23 (homolog of yeast Rad23) as well as several components of the 26S proteasome. Interestingly, we also found three ubiquitin ligases and two ubiquitin conjugating enzymes of unknown function to be associated with dHrd1 (data not shown). Whether dHrd1 contributes to degradation mediated by these enzymes or whether they modulate dHrd1 function remains to be determined. To the best of our knowledge, these studies mark the first ubiquitin ligase complex characterized at the molecular level in *Drosophila*.

RNAi-mediated knockdown of dHrd1-associated proteins including dSel1, dUbc7, dNpl4, dUfd1, Ter94, dUbiquilin, and dHerp blunted the sterol-induced ERAD of reductase in S2 cells (Fig. 1). Similarly, knockdown of genes encoding several of these proteins also blunted another aspect of reductase ERAD, namely dislocation of the protein into the cytosol for proteasomal degradation (9). Consistent with results in mammalian cells (9), reductase dislocation required the prior ubiquitination of reductase as indicated by the inhibition of the reaction in dHrd1 and dSel1 knockdown cells (Fig. 2). In addition, cytosolic dislocation of reductase required the *Drosophila* VCP/p97 homolog Ter94 (Fig. 2). Knockdown of dHerp also blunted reductase dislocation; however, the precise role for dHerp in reductase ERAD remains to be determined. Notably, knockdown of dUbiquilin failed to inhibit reductase dislocation (Fig. 2). dUbiquilin is a homolog of the yeast protein Dsk2p, which combines with another protein called Rad23 to shuttle ubiquitinated proteins to the proteasome for degradation. Thus, the possibility exists that dUbiquilin participates in delivery of cytosolic reductase to proteasomes.

Considering the successful reconstitution of reductase ERAD in S2 cells, we extended our studies to Insig-1, whose ERAD is subjected to lipid-mediated regulation in mammalian cells (17, 18). Remarkably, we found that the selectivity of Insig-1 ERAD was preserved in the *Drosophila* system. For example, Insig-1 but not its highly related isoform Insig-2, was subjected to proteasome-mediated ERAD in S2 cells (Fig. 3A); polyubiquitinated forms of Insig-1 were identified in the presence of the proteasome inhibitor MG-132 (Fig. 3B). Similar to the situation in mammalian cells, the ERAD of Insig-1 was inhibited by sterols in S2 cells through a mechanism that required coexpression of the cholesterol-sensing SREBP escort protein, Scap (Fig. 3C). Finally, Insig-1 ERAD was inhibited by the unsaturated fatty acid oleate through a mechanism that did not require the coexpression of Scap (Fig. 3D). Thus, reductase and Insig-1 are subjected to lipid-regulated ERAD in both *Drosophila* and mammalian systems, indicating that mechanisms underlying their selection for ERAD are highly conserved across species.

Further investigation revealed that dTeb4, the *Drosophila* homolog of the membrane-bound yeast ubiquitin ligase Doa10 and mammalian Teb4 (28), was required for Insig-1 ERAD in S2 cells (Fig. 4A). This finding is surprising considering that *i*) Insig-1 presumably binds to dHrd1 as it bridges the ubiquitin ligase to reductase in sterol-treated cells; and *ii*) the same ubiquitin ligase, gp78, is required for ubiquitination and degradation of both reductase and Insig-1 in mammalian cells (19, 42). Overexpression of dHrd1 failed to rescue Insig-1 ERAD in dTeb4 knockdown cells (Fig. 4C); however, the reaction was fully restored upon overexpression of another membrane-bound ubiquitin ligase, dTrc8 (Fig. 4D). This result indicates that similar mechanisms underlie selection of Insig-1 for dTrc8- and dTeb4-mediated ERAD in S2 cells. Whether this involves direct interaction between the ubiquitin ligases and Insig-1 or whether a shared factor is involved in selection of Insig-1 for ERAD is not clear, and requires the molecular characterization of dTrc8 and dTeb4 ubiquitin ligase complexes.

In the absence of RNAi, dHrd1 overexpression blocked ERAD of Insig-1 (Fig. 5A), suggesting that the ubiquitin ligase sequesters components of the ERAD pathway required for dTeb4-mediated ubiquitination of Insig-1. To evaluate this possibility, we examined a role for various dHrd1 complex components in Insig-1 ERAD. The results show that several dHrd1 complex components including dUbc7, dUbxd8, dHerp, Ter94, dUfd1, and dNpl4 are required for ERAD of both Insig-1 (Fig. 5B) and reductase (Fig. 1). Interestingly, the ERAD of reductase has a specific requirement for dSel1 and dUbiquilin (Fig. 1), while Insig-1 ERAD specifically requires dUbc6 (Fig. 5B). We previously found that dDerlin 2/3 was not required for reductase ERAD in S2 cells (21), but the protein is required for the ERAD of Insig-1 (Fig. 5B). These differences likely reflect the differential actions of dTeb4 and dHrd1 in the ERAD of reductase and Insig-1 and indicate they may occur through distinct pathways. This notion is supported by results of experiments shown in Figs. 2 and 6A, which evaluate a role for dHrd1 complex components in cytosolic dislocation of reductase and Insig-1. Knockdown of dHrd1 and dSel1, which mediate reductase ubiquitination, blocked both the sterol-accelerated ERAD and sterol-induced cytosolic dislocation of reductase (Figs. 1, 2). Knockdown of the ubiquitin ligase dTeb4 or the ubiquitin-conjugating enzyme dUbc6 blocked Insig-1 ERAD, but not its cytosolic dislocation (Fig. 6A). Instead, knockdown of genes encoding proteins involved in postubiquitination steps of ERAD including Ter94, dUfd1, and dNp14 inhibited the reaction. Similar results were obtained in S2 cells treated with PYR-41, an inhibitor of ubiquitin-activating enzymes (40). PYR-41 treatment stabilized Insig-1 in membranes and led to the accumulation of the protein in the cytosol (Fig. 6B), indicating that dislocation of Insig-1 into the cytosol precedes ubiquitination.

Considered together, the current results establish that mammalian reductase and Insig-1 are degraded through distinct mechanisms in *Drosophila* S2 cells. The reductase appears to become ubiquitinated through a reaction that requires the ubiquitin ligase dHrd1, its cofactor dSel1, and the ubiquitin-conjugating enzyme dUbc7. This ubiquitination marks reductase for cytosolic dislocation, which is likely mediated by the ATPase Ter94, its membrane receptor dUbxd8, and the cofactors dUfd1 and dNpl4. Dislocation of reductase also appears to be modulated by the *Drosophila* homolog of Herp, and dUbiquilin mediates steps in reductase ERAD following dislocation of the protein into the cytosol. In contrast to reductase, Insig-1 appears to become dislocated into the cytosol prior to dTeb4-mediated ubiquitination. Importantly, this dislocation requires dDerlin-2/3, a member of the Derlin family of polytopic membrane proteins in yeast and mammals that play a key role in the ERAD of both soluble and membrane-bound ERAD substrates (43, 44). It is worth noting that although RNAi-mediated knockdown of dUbc6 or dUbc7 significantly blunted the ERAD of Insig-1 (Fig. 5B), dUbc7 but not dUbc6 appears to be required for cytosolic dislocation of Insig-1 (Fig. 6A). A likely explanation for this discrepancy is that an unknown *Drosophila* ubiquitin ligase combines with dUbc7 in the ERAD of Insig-1 in S2 cells. We postulate that this putative ubiquitin ligase directs Insig-1 through a dHrd1-like ERAD pathway in which the substrate becomes ubiquitinated prior to its cytosolic dislocation. Thus, important directions for future studies include the identification of the alternative ubiquitin ligase required for Insig-1 ERAD, examining the mechanism through which Insig-1 is selected for ERAD and dislocated into the cytosol of S2 cells; determining how Insig-1 is solubilized in the cytosol prior to ubiquitination; determining whether reductase is fully extracted from S2 cell membranes prior to cytosolic dislocation; and determining whether reductase and Insig-1 are degraded through distinct mechanisms in mammalian cells. Successful completion of these studies will provide key insights into mechanisms that control lipid homeostasis and mechanisms for the ERAD of integral membrane proteins.
